# Data-Driven Elucidation of Flavor Chemistry

**DOI:** 10.1021/acs.jafc.3c00909

**Published:** 2023-04-27

**Authors:** Xingran Kou, Peiqin Shi, Chukun Gao, Peihua Ma, Huadong Xing, Qinfei Ke, Dachuan Zhang

**Affiliations:** † Collaborative Innovation Center of Fragrance Flavour and Cosmetics, School of Perfume and Aroma Technology, Shanghai Institute of Technology, Shanghai 201418, China; ‡ Laboratory for Physical Chemistry, ETH Zürich, 8093 Zürich, Switzerland; § Department of Nutrition and Food Science, University of Maryland, College Park, Maryland 20742, United States; ∥ CAS Key Laboratory of Computational Biology, Shanghai Institute of Nutrition and Health, University of Chinese Academy of Sciences, Chinese Academy of Sciences, Shanghai 200031, China; ⊥ National Centre of Competence in Research (NCCR) Catalysis, Institute of Environmental Engineering, ETH Zürich, 8093 Zürich, Switzerland

**Keywords:** bioinformatics, cheminformatics, machine learning, database, active ingredients

## Abstract

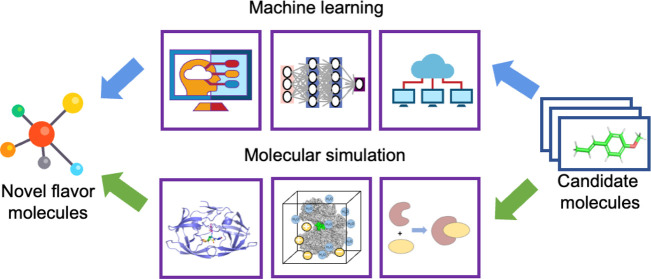

Flavor molecules are commonly used in the food industry to enhance product quality and consumer experiences but are associated with potential human health risks, highlighting the need for safer alternatives. To address these health-associated challenges and promote reasonable application, several databases for flavor molecules have been constructed. However, no existing studies have comprehensively summarized these data resources according to quality, focused fields, and potential gaps. Here, we systematically summarized 25 flavor molecule databases published within the last 20 years and revealed that data inaccessibility, untimely updates, and nonstandard flavor descriptions are the main limitations of current studies. We examined the development of computational approaches (e.g., machine learning and molecular simulation) for the identification of novel flavor molecules and discussed their major challenges regarding throughput, model interpretability, and the lack of gold-standard data sets for equitable model evaluation. Additionally, we discussed future strategies for the mining and designing of novel flavor molecules based on multi-omics and artificial intelligence to provide a new foundation for flavor science research.

## 1 Introduction

Flavor molecules have a long history of use in food products for enhancing nasal sensations and improving taste perceptions to stimulate the appetites of consumers.^1^ Beyond their key roles in defining taste and smell, some flavorings (e.g., vanillin) can increase the shelf life and stability of food products and improve their texture and appearance.^2^ In the pharmaceutical industry, the addition of flavoring agents, such as cetirizine hydrochloride and famotidine, is used to mask the unpleasant odor and taste of various drugs.^3^ Despite their recognized importance and wide application in industries, evidence suggests that certain flavor substances pose potential health risks.^4,5^ For example, some artificial sweeteners have been associated with colitis, obesity and its related comorbidities, and metabolic dysregulation.^6^ Diacetyl, a butter-flavoring compound used in plant bakeries, has been linked to increased rates of bronchiolitis obliterans, while monosodium glutamate has been linked to obesity, metabolic disorders, neurotoxic effects, and reproductive organ damage.^7^ Moreover, methyl *N*-acetyl anthranilate, a common natural berry flavoring, has been shown to cause phototoxicity.^8^

In an effort to address these health-associated challenges and promote reasonable applications, several databases, such as the Flavor Ingredient Library developed by the Flavor and Extract Manufacturers Association of the United States,^9^ AdditiveChem,^5^ and FlavorDB,^10^ have been constructed in the past two decades, which provide comprehensive and in-depth knowledge on flavor molecules. Despite the applications of big data in food science having been summarized in a previous review,^11^ no study has systematically evaluated available databases for flavor molecules through the assessment of data quality, focused fields, and potential gaps in information, which limits the further development of this field.

The perception of flavor arises from the interaction of biological machinery (e.g., the taste buds) and flavor molecules; thus, flavor perception can be regarded as an emergent property of a complex biochemical system.^10^ The rapid development of computational strategies, such as machine learning (ML) and molecular simulation (MS), provides us new opportunities for unveiling underground biological mechanisms of flavor perception. Using computational strategies, we can also analyze the structural characteristics of known flavor molecules and explore the interactions between perception receptors and candidate molecules to assist in the discovery of new flavorings with positive health impacts.^12^

**Figure 1:**
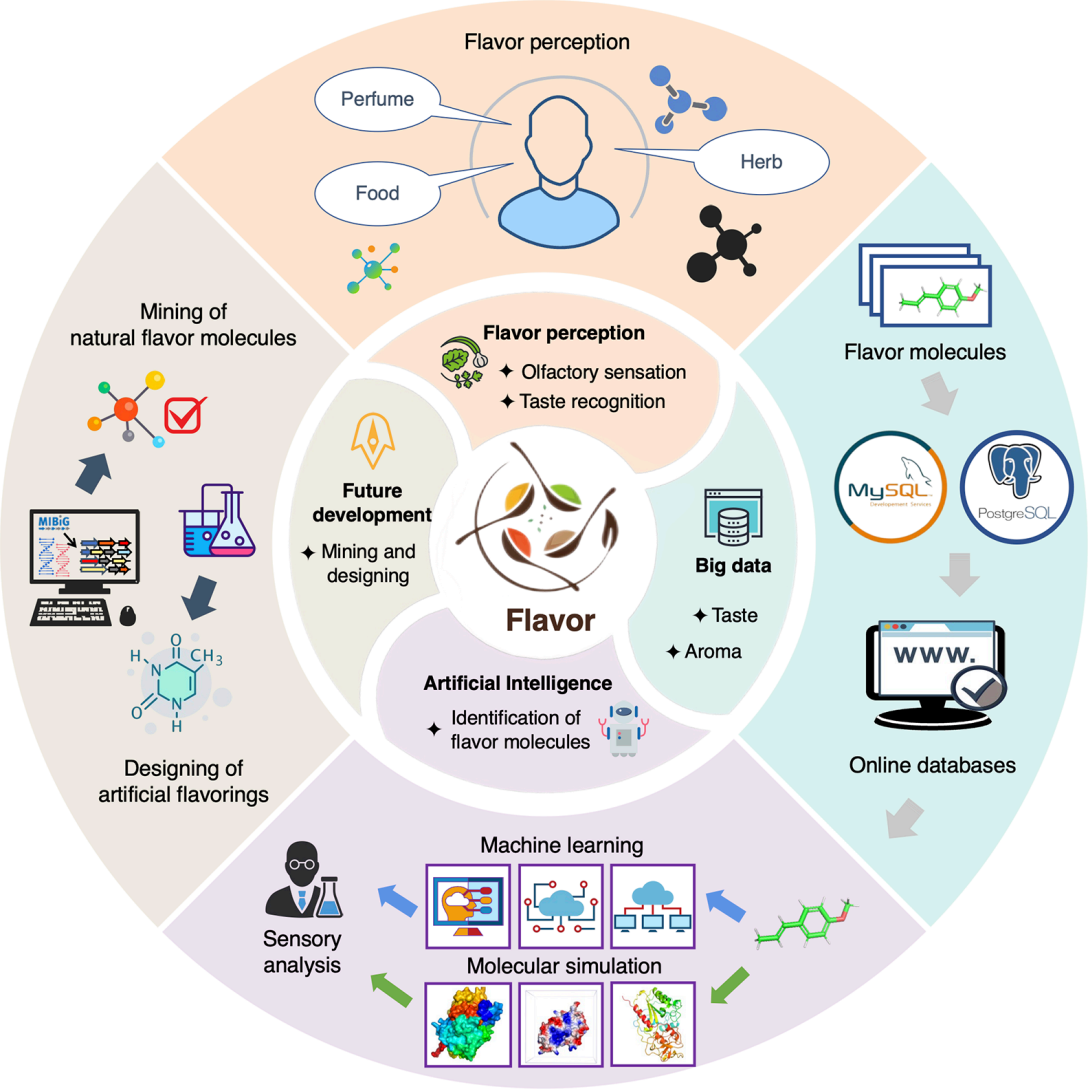
Data-driven study in flavor science. Flavor molecules in perfumes, herbs, and foods are responsible for the stimulation of human sensory perceptions. Owing to the increasing number of known flavor molecules, specialized food molecule databases were built based on data management software, such as MySQL and PostgreSQL. These databases enabled the application of computational strategies (e.g., machine learning and molecular simulation) in flavor science and, in conjunction with sensory analysis, have been successfully used to identify novel flavor molecules. With the rapid development of multi-omics and artificial intelligence, advanced computational approaches have expressed great potential in guiding the designing of artificial flavorings and the mining of natural flavor molecules.

This review summarizes databases for flavor molecules released within the last two decades and discusses the application of computational strategies for (1) identifying novel flavor molecules, (2) elucidating the molecular interaction of flavor perception, and (3) mining and designing flavor molecules based on multi-omics and artificial intelligence (Figure 1).

## 2 Flavor Molecule Databases

**Figure 2:**
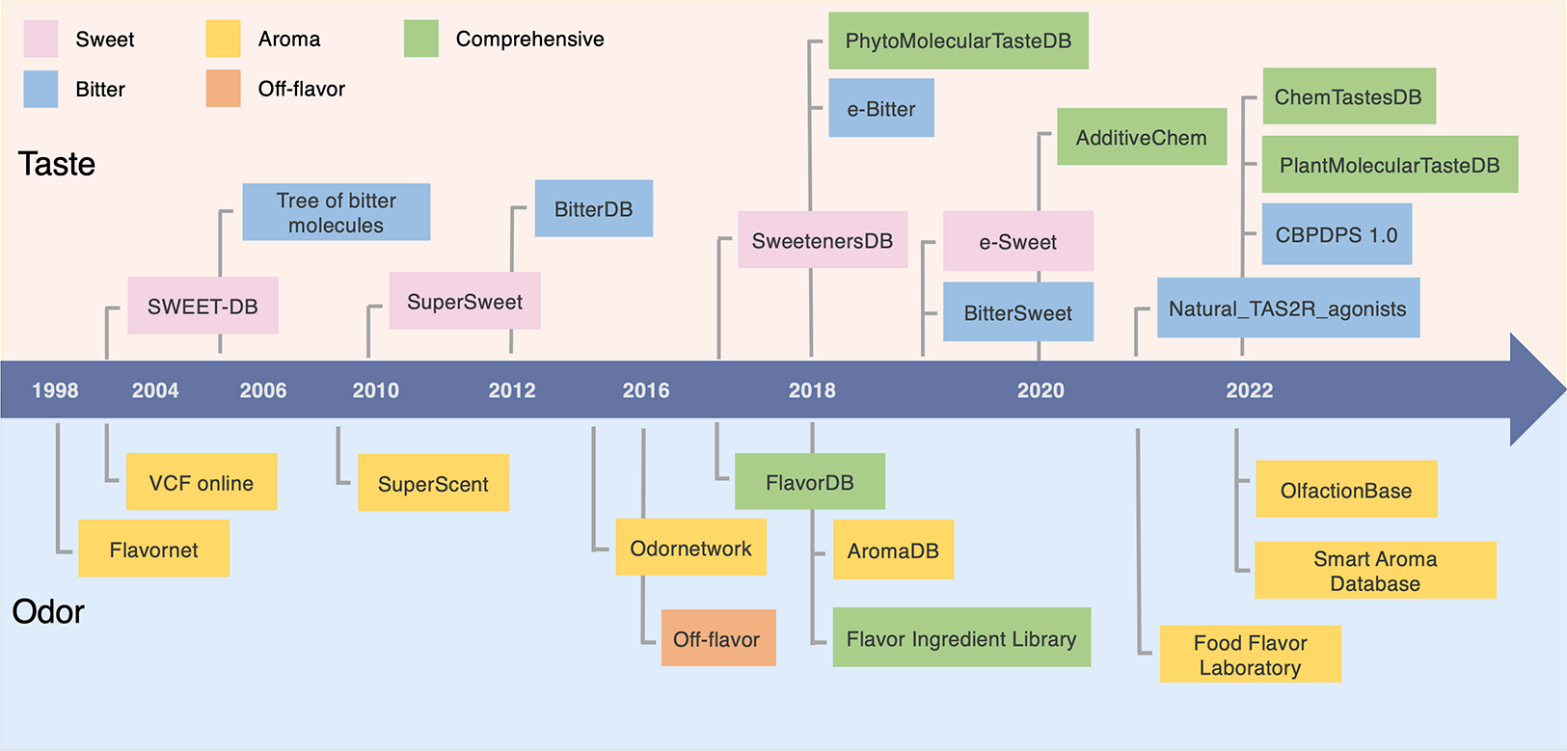
Overview of flavor databases released from 1998 to 2022. Databases containing taste molecules are presented at the top of the timeline, and databases containing aroma molecules are at the bottom. The colors represent the types of databases.

To provide an overview of known flavor molecules, we retrieved data related to flavor molecules from academic databases such as Scopus, PubMed, Web of Science, and Google Scholar. We retrieved 25 flavor molecule databases, of which 14 included taste molecules, 9 contained aroma molecules, and 2 comprised both (Figure 2 and Table 1), that contained information on molecule names, Chemical Abstract Service (CAS) registry numbers, molecular structures in a simplified molecular-input line-entry system (SMILES) format, and flavor descriptions.

**Table 1:** Summary of Flavor Molecule Databases

databases	type	URL	release date	data availability	num of molecules
SWEET-DB	sweet	http://www.dkfz.de/spec2/sweetdb/	2002	not available	
SuperSweet	sweet	http://bioinformatics.charite.de/sweet/	2011	not available	∼8000
SweetenersDB	sweet	http://chemosim.unice.fr/SweetenersDB	2017	open access	316
e-Sweet	sweet		2019	not available	530
Tree of bitter molecules	bitter		2005	not available	833
BitterDB	bitter	http://bitterdb.agri.huji.ac.il	2012, 2019	open access	1041
e-Bitter	bitter		2018	not available	1150
Natural_TAS2R_agonists	bitter	https://github.com/dipizio/Natural_TAS2R_agonists	2021	open access and downloadable	247
CBDPS 1.0	bitter		2021	open access and downloadable	1958
BitterSweet	sweet and bitter	https://github.com/cosylabiiit/bittersweet	2019	open access and downloadable	1943
PhytoMolecularTasteDB	sweet, bitter, sour, pungent, astringent, and salty		2018	open access	
AdditiveChem		http://www.rxnfinder.org/additivechem/	2020	open access	9064
PlantMolecularTasteDB	sweet, bitter, sour, pungent, umami, astringent, and salty	http://www.plantmoleculartastedb.org/	2022	open access	1527
ChemTastesDB	sweet, bitter, sour, pungent, umami, astringent, and salty	https://doi.org/10.5281/zenodo.5747393	2022	open access and downloadable	2944
Flavornet	25 types of odors	https://www.flavornet.org	1998	open access	738
VCF online	273 types of odors	https://www.vcf-online.nl/VcfHome.cfm	2002	open access	9832
SuperScent	121 types of odors	http://bioinformatics.charite.de/superscent	2009	not available	2100
Odornetwork	526 types of odors	http://odornetwork.com/network/index.html	2015	not available	3016
Off-flavor molecules			2016	open access	792
AromaDb	357 types of odors	http://bioinfo.cimap.res.in/aromadb/	2018	open access	1321
Food Flavor Laboratory	32 types of odors	http://foodflavorlab.cn/	2021	open access	171
Smart Aroma Database		https://www.shimadzu.com/an/products/gas-chromatograph-mass-spectrometry/gc-ms-system/smart-aroma-database/index.html	2022	open access and downloadable	∼500
OlfactionBase	572 types of odors	https://bioserver.iiita.ac.in/olfactionbase/	2022	open access	3985
FlavorDB		https://cosylab.iiitd.edu.in/flavordb/	2018	open access	25,595
Flavor Ingredient Library		https://www.femaflavor.org/flavor-library	2018	open access	3012

**Figure 3:**
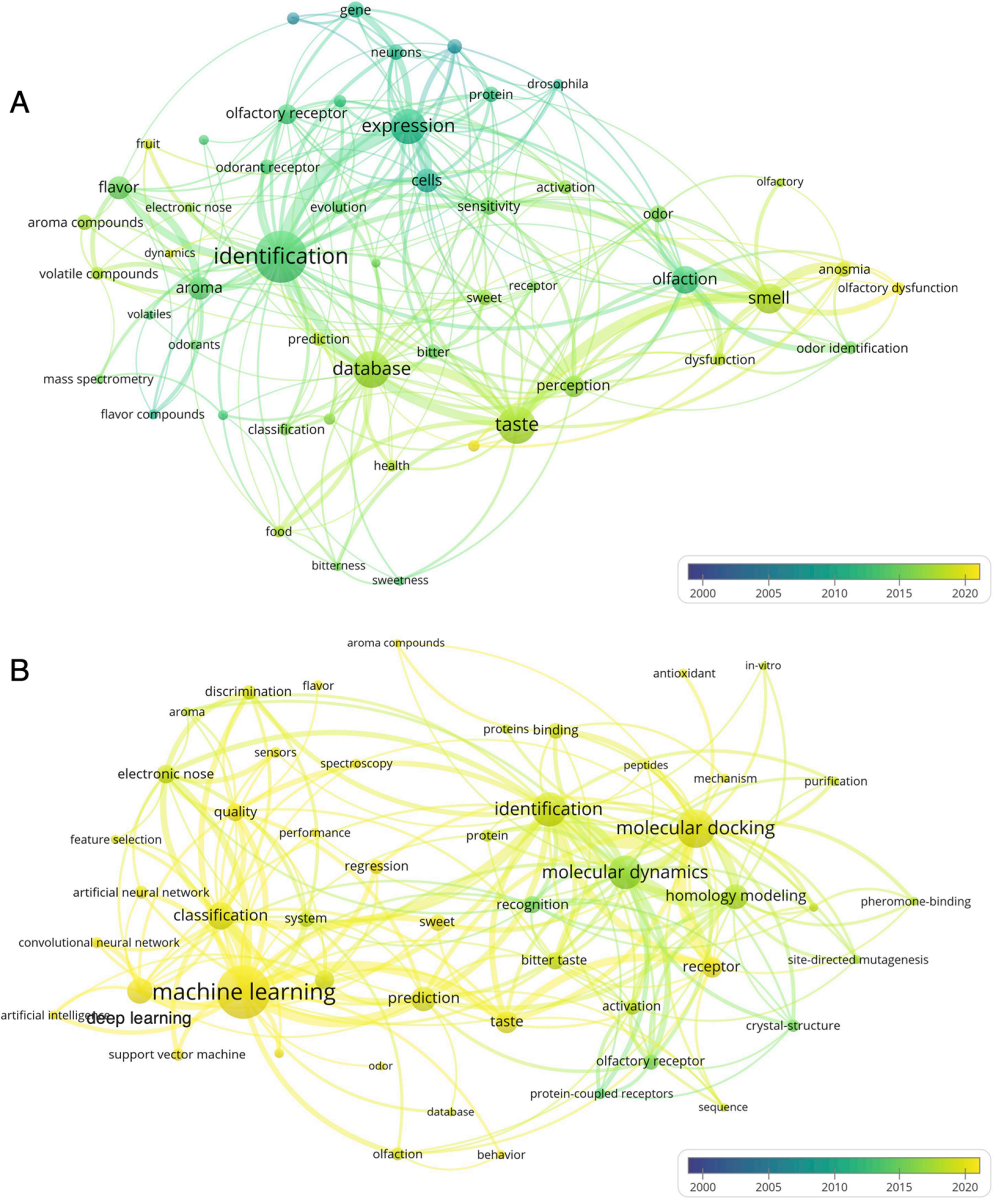
Keyword co-occurrence network of the literature related to (A) flavor databases and (B) flavor molecule identification. Each circle in the diagram represents a unique keyword. Circle size indicates the number of keyword occurrences in the literature. The color gradient from blue to yellow corresponds to the timeline (bottom right).

The keyword co-occurrence network of flavor database-related articles was constructed using VOSviewer (Figure 3A). We found that “identification” was the most frequent keyword in these publications, which implies that flavor molecule identification has been a main research focus in this field. The color and size of the circles for “database” and “taste” indicate that there are numerous databases related to “taste”, which has recently become a research hotspot. The color and size of the circles for “sweetness” and “bitterness” indicate that they are the most commonly studied taste properties. The significant links found among “identification”, “odorant receptor”, and “olfactory receptor” suggest that one primary method to identify flavor molecules is based on the interaction between molecules and receptors. The links among “genes”, “neurons”, “proteins”, and “cells” indicate the complex biological mechanism behind flavor perception. The color of the circles and links indicates the time when the corresponding literature was published. We found that terms “fruit”, “food”, “dysfunction”, and “health” appeared in recent literature, indicating that there is a growing interest in studying natural flavor molecules from food and their health effects (Figure 3A).

### 2.1 Taste Molecule Databases

Table 1 lists the taste molecule databases, including their focus, Uniform Resource Locator (URL), release date, data availability, and the number of molecules. Taste molecule databases were largely focused on sweetness and bitterness, as they are considered the most common tastes. Four databases specifically focused on sweet-taste molecules, including SWEET-DB,^13^ SuperSweet,^14^ SweetenersDB,^15^ and e-Sweet.^16^ SWEET-DB^13^ is the first publicly available sweetness database, containing several carbohydrate structures and their mass spectrometry data. SuperSweet^14^ is the largest sweet-taste database, containing more than 8000 sweet compounds and their calories, physicochemical properties, glycemic index, origin, and other information regarding molecular receptors and targets. In contrast to SWEET-DB,^13^ SuperSweet’s web server interface offers a user-friendly search and a sweet tree, which groups the sweet substances into three main families (carbohydrates, peptides, and small molecules^14^). In 2017, Chéron et al. developed SweetenersDB,^15^ a database containing 316 compounds with relative sweetness ranging from 0.20 to 225,000 to develop quantitative conformational relationship (QSAR) models. In 2019, Zheng et al. developed e-Sweet,^16^ which combines data from SuperSweet^14^ and SweetenersDB,^15^ to provide a comprehensive data set of 530 sweetener compounds and their relative sweetness values. Both SweetenersDB^15^ and e-Sweet^16^ have been utilized in machine learning to predict new sweeteners and their relative sweetness levels, providing valuable information for designing new sweeteners.

Six databases focused specifically on bitterness molecules. In 2005, Rodgers et al. constructed a phylogenetic-like tree of structural fragments to extract valuable insights from a structural database containing 833 bitter molecules.^17^ This was the first collection of bitterness molecules; however, it was not collated into a publicly available database. In 2012, Wiener et al. developed BitterDB,^18^ the first online database of bitterness molecules, which contains >550 bitter taste compounds. It also contains information on mutations in receptors influenced by bitter molecules. BitterDB received an update in 2019,^19^ which increased the number of bitterness molecules to 1041 and provided additional data on molecules’ bitterness intensity, toxicity, and interactive receptors. In 2020, Bayer et al.^20^ collected a data set of 247 natural compounds with bitter taste receptor activity, of which 138 were derived from food.^19^ In 2018, Zheng et al. developed e-Bitter,^21^ a bitterant prediction model based on a data set containing 707 bitterants and 592 nonbitterants. In 2019, Tuwani et al. developed a data set containing 918 bitter molecules and 1205 sweet molecules to comprise BitterSweet,^12^ an ML model for classifying sweet and bitter molecules. Simultaneously, Bai et al. collected 911 bitter and 1248 sweet compounds to build an ML model for predicting the bitter taste of drugs.^22^

In addition, several comprehensive databases on molecules with sour, salty, spicy, and fresh tastes have been developed, such as AdditiveChem,^5^ PhytoMolecularTasteDB,^23^ and ChemTastesDB.^24^ PlantMolecularTasteDB^25^ contains 1527 phytochemicals from 394 plants and their taste senses (e.g., bitter, sweet, sour, fresh, salty, pungent, and astringent) and anti-inflammatory properties. A unique feature of PlantMolecularTasteDB absent in other taste-focused databases consists of data on the evidence-based biological activity of the phytotastants.^25^ AdditiveChem^5^ curated >9064 types of food additives (most of which are flavorings), including information on their molecular structures, physicochemical properties, biosynthesis methods, usage specifications, risk assessment data, and related receptors. PhytoMolecularTasteDB^23^ includes plant-derived flavor molecules and details on the combination of tastes resulting from the main flavor molecules found in a medicinal plant. The list includes 431 Ayurvedic medicinal plants, 223 phytochemical classes, and 438 plant-derived molecules. ChemTastesDB^24^ contains information on 2944 verified compounds divided into nine classes, comprising the five basic tastes (sweet, bitter, umami, sour, and salty) and four additional categories: tasteless, nonsweet, multi-taste, and miscellaneous. These databases constitute novel tools for the scientific community to expand information on taste molecules and analyze the relationships between molecular structures and flavor properties.

### 2.2 Aroma Molecule Databases

In aroma molecule databases, molecule olfactory descriptions are typically named after the substance that produces the odor, such as rose fragrance, meat fragrance, and fish fragrance. Flavornet,^26^ a compilation of aroma compounds found in the human odor space, was first published in 1998 and last updated in 2004. It contains 738 odorants with their associated CAS registry numbers and 2D structures. These have been classified into 197 categories based on their odor descriptions, such as almond, cabbage, cheese, and herb; however, keywords of molecule odors cannot be used to search this database. The development of SuperScent^27^ in 2009 addressed this issue, offering a variety of search options based on chemical names or the molecular structures of odorants. In addition, it contains 2147 volatile compounds classified according to their sources, functions, and odor groups, as well as their chemical properties and commercial information. Unfortunately, it has not been consistently maintained. Odornetwork^28^ is another database that is no longer being maintained. Kumar et al. established 526 sensory descriptions and 3016 corresponding flavor molecules from perfume, food, and agricultural and pharmaceutical industries.^28^ In 2016, Ueda et al. developed a database of 792 molecules with unpleasant odors, including alcohols, aldehydes, carboxylic acids, esters, ethers, and hydrocarbons using gas chromatography–mass spectrometry.^29^ Kumar et al. developed AromaDB in 2018,^30^ a database providing 1321 essential oil/aroma compounds from 166 commercially used plants and their bioactivities. Moreover, the database includes additional information regarding the interaction of aroma molecules with proteins/genes. This helped to reveal the action mechanisms of aroma molecules and their potential use in treating diseases. The Food Flavor Laboratory Database was developed in 2021, providing information on 171 flavor compounds, including their CAS numbers, chemical structures, aroma thresholds, and descriptions. OlfactionBase^31^ contains extensive coverage of 5109 odorants, 2067 olfactory receptors, and 874 OR-odorant pairs. In addition, it contains information on 2871 odorant-binding or pheromone-binding proteins from 190 species.

In addition to academic databases, several commercial databases are available such as the Smart Aroma Database and Volatile Compounds in Food (VCF) online database. The Smart Aroma Database contains information on >500 compounds that contribute to aroma, enabling the objective evaluation and analysis of aroma compounds using gas chromatography–tandem mass spectrometry. The VCF online database contains 9832 volatile substances in food products and their odor descriptions and aroma thresholds. In addition, several databases contain both aroma and taste properties of molecules, including the FEMA Flavor Ingredient Library^9^ and FlavorDB.^10^ The Flavor Ingredient Library is a database of 3012 flavor substances that includes safety assessments and publications. It provides an indispensable resource for researchers, media, and consumers seeking information on flavor ingredients whose safety has been determined to be generally recognized as safe (GRAS) by the independent FEMA Expert Panel. FlavorDB^10^ contains 25,595 flavor molecules, including 2254 natural molecules, 13,869 synthetic molecules, and 9472 molecules of an unknown origin. It divides flavor molecules into 31 categories, containing records of molecular, sensory, absorption, distribution, metabolism, elimination, toxicity properties, literature sources, and flavor characteristics. It may be used to find molecules matching a desired flavor or structure, explore molecules of an ingredient, discover novel food pairings, determine the molecular essence of food ingredients, and associate chemical features with a flavor.

### 2.3 Current Limitations and Future Perspectives of Flavor Molecule Databases

These data are helpful for researchers studying flavor profiles and the mechanisms of action between taste and olfactory receptors and provide chemists with convenient, high-quality data resources. However, several issues need to be addressed. For example, ∼70% of databases are not downloadable or must be requested by the authors. This limits data reuse and makes assessing data quality and integrity difficult. Furthermore, certain databases such as SweetDB^13^ and SuperSweet^14^ are currently unavailable, and those available are not regularly updated post publication; thus, they are unsuitable for use in current research. Another consideration is that taste molecule data have been mostly derived from sweet and bitter molecules, with >50% of taste molecule databases focused on bitterants and sweeteners. As a result, other taste sensations have received less attention. This highlights the need to further annotate molecules with sour, salty, and spicy tastes in publications and experimental records. Annotating odor categories of volatile molecules is more challenging than assigning taste categories. Most odor molecule databases divided the odors into hundreds of classes based on the substance that produces the smell, which has led to nonstandard odor names. Meanwhile, the flavor threshold and content in natural resources of most flavor molecules are yet to be included in any databases, which may limit their application in industries. These issues should be considered and addressed in future studies.

To facilitate further data reuse, we collected the known flavor molecules from these databases and subsequently removed redundancies and molecules with amphibolous descriptions (e.g., sweet-like and nonsweet). Finally, 8982 molecules with a known taste and 5046 with a known aroma were obtained, which are provided in a GitHub repository along with this paper.

## 3 Screening and Designing of Flavor Molecules Based on Computational Strategies

Comprehensive data on flavor molecules provide a new opportunity for identifying novel flavor molecules based on data-driven computational strategies. The size and color of the circles representing the keywords “taste”, “aroma”, “machine learning”, “molecular dynamics”, and “molecular docking” indicate that molecular simulation and machine learning have been widely used in flavor molecule research (Figure 3B). The links among the keywords “identification”, “homology modeling”, and “receptor” indicate the typical pipeline for identifying novel flavor molecules based on the interaction between receptors and molecules (Figure 3B). Machine learning is usually used for “classification” and “regression” tasks in flavor research, with algorithms, including random forest (RF), support vector machines (SVM), and convolutional neural networks (CNN) (Figure 3B), for example, regression prediction of the aroma thresholds and classification prediction of taste class of molecules.

### 3.1 Molecular Simulation

**Figure 4:**
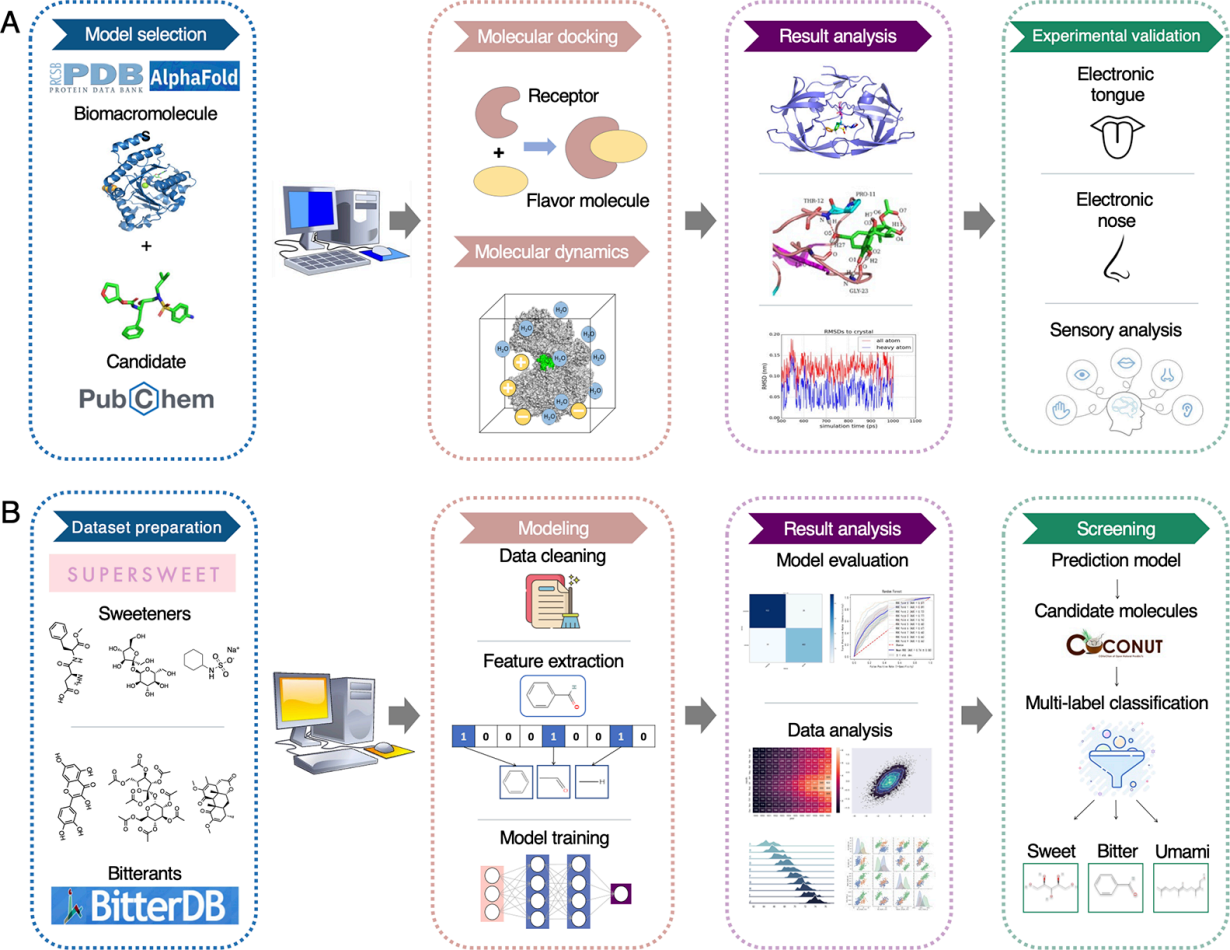
Schematics of computational strategies for mining novel flavor molecules. (A) Schematic of molecular simulation, including data preparation, simulation, result analysis, and experimental validation. (B) Schematic of molecular machine learning, including data set preparation, modeling, result analysis, and screening.

Molecular dynamics and molecular docking are common MS methods that are used in flavor studies.^32,33^ Molecular dynamics is a computational simulation of a complex biological system that describes motions, interactions, and dynamics at the atomic level.^34^ This is achieved by choosing a “force field” representing all the interatomic interactions and integration of Newtonian equations, which provide the position and speed of atoms over time.^34^ It has been increasingly used to explore mechanisms of interaction and conformational relationships between flavor molecules and receptors (Table 2). Molecular docking is a technique based on the lock-and-key theory.^35^ By computing the intermolecular interactions between the flavor molecules and receptors, it predicts their probable binding modes. Common types of intermolecular interactions include van der Waals forces, electrostatic forces, hydrophobic interactions, and chemical bonds.^36^ By minimizing these energies, the most stable binding conformation will be identified.^36^ The results of molecular dynamics and molecular docking improve our understanding of the flavor properties of molecules and serve as guidelines for downstream experimental analyses^37^ (Figure 4A).

**Table 2:** Summary of Application of Molecular Simulation and Machine Learning in Flavor Science^a^

date	strategy	target flavor	task	algorithm and software	experimentally verified	num of molecules for modeling	data source	code availability
2016	MS	bitter	characterize the interaction of steviol glycosides with bitter taste receptors	Autodock Vina and NAMD	×		PDB and PubChem	
2018	MS	sweet	characterize the interaction of natural sweeteners with human sweet taste receptors	NAMD	×		PDB, UniProtKB, and PubChem	
2021	MS	umami	characterize the interaction of peptides with umami receptors	GROMACS and Discovery Studio	√		homology modeling	
2021	MS	sweet	study the synergic effect of various sweetener blend combinations of artificial and natural sweeteners	NAMD and GROMACS	×			
2021	MS	umami	characterize the interaction of umami peptides with umami receptors	Discovery Studio	√		homology modeling and literature	
2022	MS	sweet	analyze the interaction of sweeteners with receptors	Schrödinger Glide, NAMD, CHARMM, and SwissParam	×		homology modeling and PubChem	
2022	MS	umami	analyze the interaction mechanism between umami taste peptides and umami taste receptors	Discovery Studio	√		homology modeling	
2021	MS	odor	identify potential olfactory modulators	Autodock Vina, SYBYL-X, and GROMACS	×		PDB and literature	
2021	MS and ML	sweet and bitter	identify sweeteners	RF and Autodock	×	2400	literature	×
2023	ML	astringency	predict the astringency thresholds and astringency types	GPR, SVR, RF, GBDT, GNB, SVM, KNN, and SGD	×	64	literature	×
2022	MS and ML	umami	identify umami molecules	PCA, SVM, RF, Autodock, and Discovery Studio	√	15,215	SWEET-DB and BitterDB	√
2002	ML	sweet	predict sweetness value of molecules	MLR	×	103	literature	×
2013	ML	sweet	predict sweetness value of molecules	MLR and SVM	×	320	literature	×
2017	ML	sweet	predict sweetness value of molecules	SVM and RF	×	316	SweetenersDB	×
2018	ML	sweet	predict sweetness value of molecules	ANN	×	487	literature	×
2018	ML	sweet	predict sweetness value of molecules	PLS	√	320	literature	×
2019	ML	sweet	predict sweetness value of molecules		×	315		×
2020	ML	sweet	predict sweetness value of molecules	RF, SVM, AdaBoost, and KNN	√	316	SweetenersDB	×
2022	ML	sweet	predict sweetness value of molecules	DT, KNN, SVM, RF, XGBoost, GBT, and PLS	×	3324	literature	√
2016	ML	bitter	predict bitterant–TAS2R interactions	SVM	√	540	BitterDB	×
2017	ML	bitter	identify bitter taste molecules	AdaBoost	√	691	BitterDB	√
2021	ML	bitter	predict bitterness value of molecules	XGBoost	×	180	BitterDB	×
2021	ML	bitter	identify bitter taste molecules	RF and XGBoost	×	2367	BitterDB	×
2022	ML	bitter	identify bitter peptides	SVM, RF, LGBM	×	320	iBitter	×
2016	ML	sweet and bitter	classify sweet taste, bitter taste, and tasteless molecules	KNN	×	1074	literature	×
2018	ML	sweet and bitter	classify bitter and sweet taste molecules	RF	×	1202	SuperSweetDB, BitterDB	×
2019	ML	sweet and bitter	classify bitter and sweet taste molecules	RF, RLR, and AdaBoost	×	4462	literature	√
2021	ML	sweet, bitter, and sour	classify sweet, bitter, and sour molecules	RF	×	4970	SuperSweet, BitterSweetForest, BitterDB, and ChEMBL	×
2016	ML	odor	predict odor impression utilizing the mass spectra of molecules	ANN	×	121	NIST Chemistry WebBook	×
2017	ML	odor	predict human olfactory perception	RF and RLM	√	338	literature and public data	√
2018	ML	odor	computer-aided molecular design	CNN	√	480	literature and public data	×
2018	ML	odor	predict the odor character of molecules	ANN	×	999	NIST Chemistry WebBook	×
2019	ML	odor	identify the relationships between odor descriptions and chemical structures		√	1689	PubChem and literature	×
2020	ML	odor	predict odor perception of molecules	RF, SVM, GBDT, AdaBoost, XGBoost, and KNN	√	480	literature	×
2021	ML	odor	identify the primary odor perceptual descriptors	Ridge, Lasso and Elastic Net regression	√	144	literature	×

a Note: MS, molecular simulation; NAMD, nanoscale molecular dynamics; CHARMM, Chemistry at HARvard Macromolecular Mechanics; PCA, principal component analysis; SVM, support vector machine; RF, random forest; MLR, multiple linear regression; KNN, K-nearest neighbor; ANN, artificial neural network; RLM, regularized linear models; CNN, convolutional neural networks; PLS, partial least-squares; RLR, logistic regression; GBDT, gradient boosting decision tree; GPR, Gaussian process regression; SVR, support vector regression; GNB, Gaussian naive bayes; SGD, stochastic gradient descent.

In MS of flavor perception, the desired proteins are the receptors related to flavor perception that are distributed on the surfaces of tongue and nose. In the mammalian taste system, the heterodimer of taste receptor type 1 members 1/3 (T1R1/T1R3) functions as an umami taste receptor, taste receptor type 1 members functions as bitter taste receptosr, and T1R2-T1R3 functions as a sweet taste receptor.^38^ The transient receptor potential channel members, polycystin 1 like 3 (PKD1L3) and PKD2L1, are candidates for sour taste receptors.^39^ Salty taste receptors primarily include the epithelial sodium channel, sodium-specific salt taste receptor, nonspecific salt taste receptor, and a taste variant of the vanilloid receptor-1 nonselective cation channel.^40^ Unlike in taste perception, aroma molecules do not specifically bind to an olfactory receptor. Conversely, an aroma molecule can bind to several olfactory receptors with varying affinities depending on their physicochemical properties.^41^ Upon binding to the odor receptor, structural changes of olfactory receptors activate olfactory G proteins. The G proteins activate the lytic enzyme, adenylate cyclase, to convert ATP to cyclic AMP (cAMP). Cyclic nucleotide-gated ion channels in the cells open in response to cAMP, allowing calcium and sodium ions to enter the cell, depolarizing olfactory receptor neurons, and transmitting information to the brain.^42^

Recently, MS has been commonly used to study the interactions between receptors and small molecules to identify novel molecules with potential flavor properties (Table 2). Several studies have focused on sweetness perception and the synergic effects of sweeteners.^43−45^ For example, Acevedo et al. developed a comparative model of hT1R2 and hT1R3 subunits to identify their interactions with natural, noncaloric sweeteners, including sweet proteins and glycosylated terpenoids, at the molecular level.^38^ Jang et al. conducted MS using predicted structures of the TAS1R2/1R3 heterodimer to analyze the synergic effects of various sweetener blend combinations of natural and artificial sweeteners.^44^ To study interactions between receptors and sweeteners, Miao et al.^43^ chose eight sweeteners by molecular docking to develop sweetener-T1R2-membrane systems to guide the designs of novel and healthy sweeteners. Subsequently, Acevedo et al. characterized the interaction of steviol glycosides with bitter taste receptors (hT2R4 and hT2R14) at the molecular level, leading to a better understanding of the natural sweeteners’ off-flavor perception in food products.^46^

In addition, MS has been used to screen and design flavor peptides.^47,48^ For example, Zhang et al. used molecular dynamics to analyze the interactions between peptides and umami receptors and identified five novel peptides with stronger umami intensity than monosodium glutamate.^47^ Using molecular docking, Gao et al. identified several novel umami peptides and found that Phe527 on T1R1/T1R3 was the key binding site, and hydrogen bonding, electrostatic interactions, and hydrophobic interactions were the main binding forces.^48^ Moreover, MS has been successfully used to guide the designing of odor molecules. In olfactory pathways, the odorant binding protein 1 (OBP1) is the main receptor for odor recognition on the malarial vector; thus, it can be used to modulate mosquito behavior and develop new attractants or repellents.^49^ Using MS and hierarchical virtual screening, Bomfim et al. successfully identified a modulator for *Anopheles gambiae* OBP1, indicating the potential application of MS in molecular screening and designing.^49^

### 3.2 Machine Learning

ML is an interdisciplinary subject involving statistics, convex analysis, probability theory, and approximation theory.^50^ ML fits mathematical/statistical functions on given data sets and can be subsequently applied to predict the flavor properties of compounds; thus, it is used for high-throughput screening of novel flavor molecules. Current ML-based flavor studies can be divided into two main categories: regression and classification (Figure 4B).

For the regression task, researchers have used various fingerprints of flavor molecules as the input and flavor properties (e.g., sweetness values) as the output in ML models, which could be considered a type of QSAR model.^51^ In 2002, Barker et al. developed the first QSAR model for sweetness value prediction. The model was developed using multiple linear regression (MLR) and parameters generated from molecular field research on 103 sweeteners and their sweetness levels from the literature.^52^ However, molecular field-based descriptors limit the model’s application domain to molecules with a similar molecular scaffold. Subsequently, several algorithms and descriptors were used to improve the performance of ML models.^15,53,54^ For example, Zhong et al.^53^ collected information from the literature on 320 sweeteners and developed an ML model for sweetness value prediction based on two algorithms, namely, SVM and MLR, and 1235 descriptors were calculated with ADRIANA.Code. Owing to the more comprehensive data set and state-of-the-art algorithms and descriptors, the test set achieved *R*^2^ = 0.882. This result is a vast improvement compared with that of Barker et al.^52^

The establishment of SweetenersDB^15^ and BitterDB^19^ largely prompted the development of ML-based sweetness prediction. Based on data from SweetenersDB,^15^ Bouysset et al. developed a new ML model and implemented a freely accessible web server for sweetness prediction.^55^ Using this web server, they successfully identified three natural compounds that activated the T1R2/T1R3 expressed in human embryonic kidney cells. Margulis et al. developed ML models based on BitterDB^19^ to predict the bitterness of compounds, thereby guiding drug design.^56^ Their results suggested that ∼25% of drugs are predicted to be very bitter, with a higher prevalence (∼40%) in COVID-19 drug candidates and microbial natural products.^56^ In addition, ML has been successfully used for odor prediction.^41,57^ Keller et al.^41^ launched an international competition in which several teams observed the smell of a molecule and how it was perceived by humans. The resulting models accurately predicted odor intensity and pleasantness, in addition to successfully predicting 8 among 19 odors, including garlic, fish, sweet, fruity, burnt, spices, flower, and sour.^36^

Binary classification is another ML task used in flavor studies; for example, it can be used to determine whether a molecule has a bitter taste. Dagan-Wiener et al. developed the ML classifier BitterPredict^58^ to predict the bitterness of compounds based on their molecular structures. Using BitterPredict, they found that 77% of natural products are bitter with certainty.^58^ This tool will help food scientists to identify whether certain ingredients are likely to be bitter and if taste masking is necessary. Predicting compound bitterness, therefore, by adopting taste-masking and flavor correction strategies is also crucial for solving the problem of drug compliance in children. Bai et al. developed an ML model, “Children’s Bitter Drug Prediction System”, which predicts whether a medicine tastes bitter.^22^ Aroma property prediction also could be considered a classification task. Licon et al. developed a method based on a subgroup discovery algorithm to discriminate perceptual qualities of smells based on physicochemical properties.^59^ They performed experiments on 74 olfactory qualities and demonstrated that the generation of rules linking chemistry to odor perception was possible, providing a new understanding of the relationship between stimuli and olfaction perception.^59^

However, these ML classification models are limited by the availability of negative samples (e.g., non-sweet and non-bitter molecules) owing to the lack of reports in the literature. To address this issue, several studies have proposed different strategies based on known data to predict the sweetness or bitterness of a molecule.^12,60,61^ Rojas et al. collected sweet, tasteless, and bitter molecules from literature to develop a classification model using the K-nearest neighbor algorithm.^60^ Banerjee et al. collected 517 sweeteners from SuperSweet^14^ and 685 bitter compounds from BitterDB^19^ and developed an ML model based on the RF algorithm.^61^ They used the model to screen the sweet or bitter tastes of the natural compounds from the SuperNatural II Database and found 197 sweet-predicted compounds and identified 3865 compounds as bitter with a confidence scores threshold of 0.95.^61^ Fritz et al. implemented ML models to predict three different taste end points, including sweet, bitter, and sour, which achieved an overall accuracy of 90% by 10-fold cross-validation.^62^ Chacko et al. developed ML models for predicting odor characters using several ML algorithms, such as RF, gradient boosting, and SVM, and 196 two-dimensional RDKit molecular descriptors as the models’ inputs.^63^ In addition to traditional features, such as physicochemical properties and molecular fingerprints, features extracted from mass spectra have also been used for ML modeling.^64,65^ For example, Nozaki et al. designed a novel predictive model which utilized mass spectrometry data with nonlinear dimensionality reduction and natural language processing.^65^ ML can also be utilized for the identification of flavor peptides. Jiang et al. developed iBitter-DRLF for the flavor property prediction of peptides based on sequence embedding techniques, soft symmetric alignment, unified representation, and bidirectional long short-term memory.^66^

These ML models have achieved great performance; however, recent studies have shown that a molecule can have multiple tastes or aromas (e.g., taste both “bitter” and “sweet”).^67^ Data we collected from these publicly available databases are consistent with these findings, revealing that 5% of collected molecules have multiple tastes, and 78% have multiple aromas. Thus, the task of classifying molecule flavor is more suitable to be considered a multilabel classification (generate multiple outputs) than a multiclass classification. Recently, Li et al. designed an ML model to identify the odor perception descriptors using multioutput linear regression models, which solved this issue.^68^ Several screening pipelines combining ML and MS to identify novel flavor molecules have been developed to achieve more accurate prediction. For example, Goel et al. designed a framework comprising QSAR models and molecular docking for identifying possible sweeteners from natural molecules.^69^ Xiu et al. developed an *in silico* pipeline to identify novel umami-tasting molecules in batches from SWEET-DB^13^ and BitterDB^19^ databases via principal component analysis, QSAR modeling, molecular docking, and electronic tongue analysis.^70^ They identified 18 novel umami molecules using the pipeline via an electronic tongue analysis.^70^

### 3.3 Limitations and Future Perspectives of Computational Strategies

Numerous studies have demonstrated the advantages of MS and ML for flavor molecule studies, but with limitations. For example, most studies for predicting novel flavor molecules require more experimental validation (e.g., e-nose, e-tongue, and sensory validation), which reduces their reliability. Furthermore, some previous models are not open-source; therefore, readers cannot replicate the algorithm and verify its accuracy. Meanwhile, most of these prediction models do not provide an online application programming interface. Therefore, flavor chemists without specialized knowledge of computational techniques may find these tools difficult to use.

Both MS and ML have notable limitations. MS relies heavily on high-performance computing resources, which limits its speed and throughput. To accelerate the screening process, Gentile et al. developed Deep Docking, a deep learning-assisted molecular docking software that utilizes QSAR models to approximate the docking outcome for unprocessed entries, thereby removing unfavorable molecules and accelerating the screening process.^71^ Thus, it may be better utilized for large-scale screening of potential flavor molecules. Notably, the screening of active ingredients for targeted receptors using the MS approach relies on high-quality protein structures to achieve accurate prediction and analysis. Although ∼200,000 protein structures have been solved, the high-resolution structures of some flavor-related receptors are still unavailable.^72^ However, the rapid development of protein structure prediction algorithms, such as RosettaFold^73^ and AlphaFold,^74^ and cryo-electron microscopes means that the accessibility of protein structures may no longer be a limiting factor in the future.

ML-based approaches have much higher throughput than MS; however, it has two major limitations: (1) low-level interpretability and (2) the need for large-scale training data. Despite the reputation of ML as an “uninterpretable black box”, it is still essential to understand how the model makes a prediction. Given this, algorithms such as SHapley Additive exPlanations (SHAP)^75^ and Sure Independence Screening and Sparsifying Operator (SISSO)^76^ have been proposed to “whiten” the black box by quantifying the contribution of features to the model’s predictions. SHAP explains model outputs using the classic Shapley values from game theory and their related extensions, while SISSO combines symbolic regression and compressed sensing to identify the most important features that describe the target property or function.^75,76^ Guo et al. have successfully used SHAP to analyze which descriptors have a close relationship with the astringency threshold.^77^ Moreover, the recent development of interpretable molecular ML, such as an iteratively focused graph network,^78^ has attempted to rank the contribution of each atom in compounds based on the model’s attention weights to increase the interpretability of prediction. ML relies on large-scale training data to achieve high performance. However, only a tiny fraction of known flavor molecules has been included in public data sets, most of which are scattered among numerous literature reports and have not been systematically curated.^68,79^ The lack of high-quality data sets can lead to studies being conducted using different training and testing data sets, making it difficult for readers to compare the performance of models. Thus, there is an urgent need to develop advanced text-mining algorithms to systematically extract flavor molecules and their properties from publications. In turn, this will help to create a comprehensive gold-standard data set to evaluate the performance of emerging ML algorithms for flavor property prediction in future studies.

## 4 Future Strategies for Identifying Flavor Molecules

**Figure 5:**
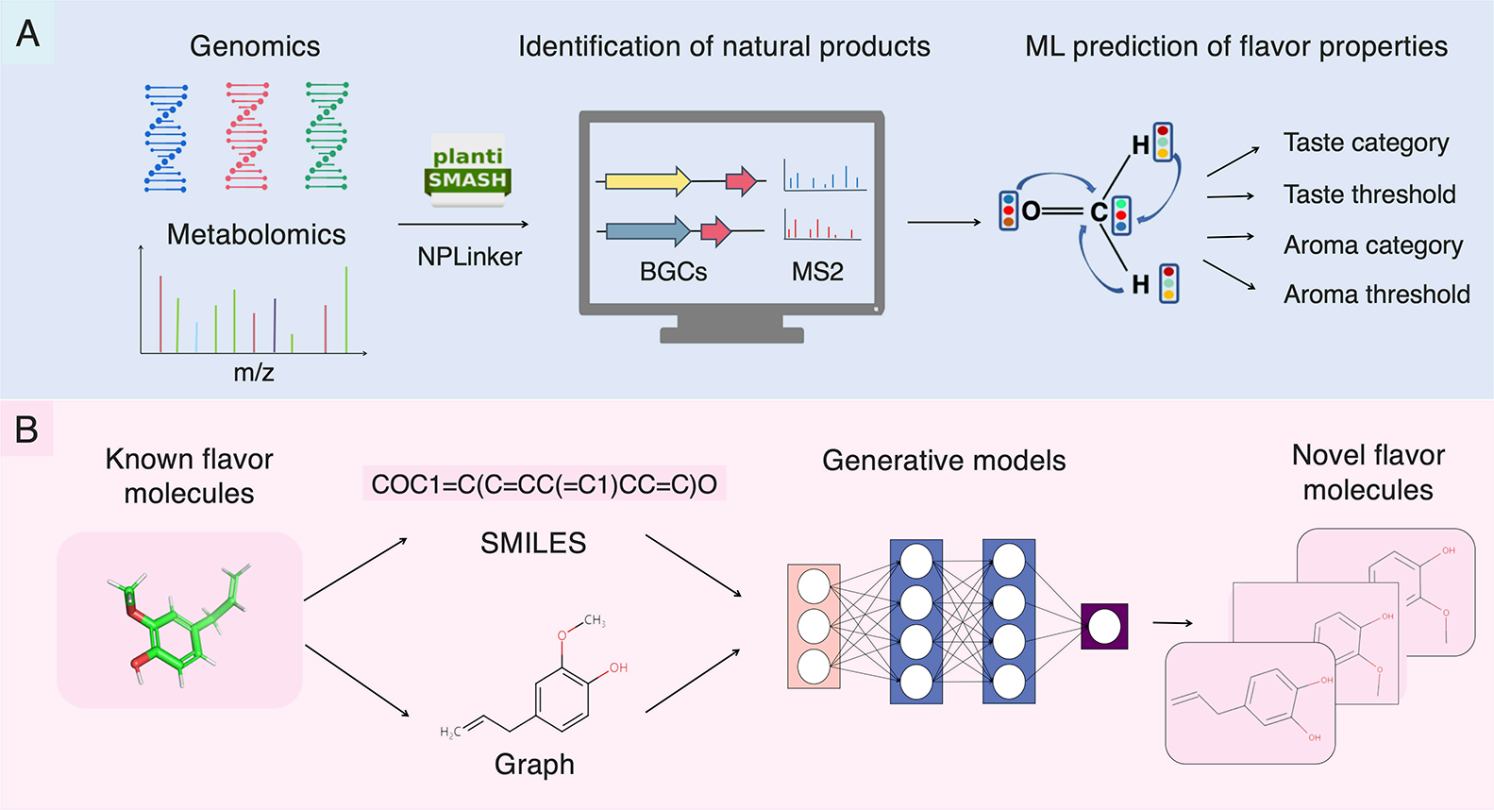
Future strategies for mining natural flavor molecules and designing artificial alternatives. (A) Schematic of mining natural flavor molecules based on multi-omics. Based on plant genome and metabolome data, novel natural products are annotated using software, such as plantSMASH and NPLinker. Machine learning models could subsequently be used to predict the flavor characteristics of these natural products to discover novel natural flavor molecules. (B) Design of artificial flavor molecules based on molecular generation. By identifying molecular presentations (e.g., string-based and molecular graphs) and functions that map a set of properties to a group of molecular structures, generative models could be used to rapidly identify diverse sets of molecules highly optimized for flavor characteristics. Note: SMILES, simplified molecular-input line-entry system.

High-throughput screening based on molecular simulation and ML enabled us to identify molecules with potential flavor properties from large-scale databases, such as COCONUT^80^ and Super Natural.^81^ However, the coverage of known molecules is still limited, with only 6% of the potential natural products evaluated.^82^ The rapid development of genomic data has revealed that plants’ biosynthesis capacity is vastly underappreciated, with millions of potential natural products awaiting discovery.^83^ Emerging computational strategies such as multi-omics and artificial intelligence provide new opportunities for mining undiscovered natural flavor molecules from food and designing purpose-built safer artificial flavorings (Figure 5).

### 4.1 Mining Natural Flavor Molecules Based on Multiomics

In plants, genes involved in specialized metabolic pathways are encoded in biosynthetic gene clusters (BGCs) contiguously on the chromosome, which facilitates the elucidation of biosynthetic pathways, thereby facilitating the identification of natural flavor molecules^83^ (Figure 5A). Several computational softwares have been developed to identify BGCs across genome sequences, including antiSMASH,^84^ PRISM,^85^ and DeepBGC.^86^ antiSMASH^84^ was first released in 2011 and updated six times over 10 years. The software identifies regions at the gene cluster level based on profile hidden Markov models (pHMMs) and aligns them to their nearest relatives of known gene clusters. Skinnider et al. developed PRISM, a platform for predicting the chemical structures of genomically encoded natural products.^85^ Using PRISM, Skinnider et al. charted secondary metabolite biosynthesis for over 10,000 bacterial genomes, revealing thousands of encoded antibiotics.^85^ DeepBGC is a deep-learning strategy to detect BGCs, which employs an RF classifier to predict the products of detected BGCs, offering an improved ability to identify new BGC classes.^86^ These tools have been widely used for elucidating novel natural products and their molecular structures from bacterial and fungal genomes. However, most known flavorings are derived from plants.^87^ To better fit the needs of plant BGC identification, Kautsar et al. developed plantiSMASH,^88^ an analysis platform for the identification of candidate plant BGCs. They applied plantiSMASH to 48 high-quality plant genomes and identified a rich diversity of candidate plant BGCs, which prompted the identification of new phytochemicals.^88^ The predictive ability of genome-based natural product annotation can be further enhanced through combination with other omics data. For example, fragmentation patterns observed in MS/MS spectra can assist in discovering metabolites and their biosynthetic genes. We could use software, such as NPLinker^89^ to link BGCs and mass spectrometry data, thereby predicting novel natural products produced by plants, and then use ML models to predict the flavor class and intensity of these newly identified molecules to identify potential natural flavorings with better flavor properties (Figure 5A).

### 4.2 Design of Artificial Alternatives Based on Artificial Intelligence

The emerging application of artificial intelligence in cheminformatics, especially molecular generation, is another promising strategy for the design of artificial flavor molecules (Figure 5B). The potential health risks of existing artificial sweeteners have encouraged scientists to design safer artificial sweeteners. Recently, *de novo* molecular design has been used in drug discovery, as it provides a reproducible methodology for artificial flavoring design. Generative models could generate molecules with desired flavor properties; therefore, these are favorable compared to designing molecules using human expertise. By identifying a function that maps a set of properties to a group of structures, generative models can rapidly identify diverse sets of molecules highly optimized for specific applications.^90^ The successful application of molecular generation largely depends on input representation and the model architecture type. To generate novel molecules with specific flavor properties, known molecules are first converted into string-based representations or molecular graphs for model training.^91^ These representations combined with the ability of deep neural networks are able to capture highly complex correlations between chemical structures and their flavor properties.

To date, molecular generative models have been used successfully for drug discovery. For example, Zhavoronkov et al. used a generative tensorial reinforcement learning model to successfully identify potent inhibitors of discoidin domain receptor 1 in 21 days, illustrating the potential of generative models for the rapid design of molecules that are synthetically feasible and possess potential innovative properties.^92^ Skinnider et al. developed DarkNPS using a generative model to determine a statistical probability distribution over unobserved structures of psychoactive substances, in turn identifying potential new psychoactive substances.^93^ Based on 1753 known psychoactive substances, they generated 8.9 million unique molecules with potential addiction. The documented successes of these practical applications encourage the use of *de novo* molecular generation for identifying novel flavor molecules. By identifying molecular presentations and functions that map a set of physicochemical properties to a group of molecular structures, generative models could rapidly predict diverse sets of molecules with highly optimized flavor characteristics (Figure 5B).

## 5 Discussion and Perspective

In this paper, we summarized 25 databases containing >14,000 unique flavor molecules (8982 molecules with known taste and 5046 with known aroma). We found that 5% of collected molecules have multiple tastes and 78% have multiple aromas, indicating the complexity of flavor perception. Although these databases have encouraged research in the field of flavor science, data in ∼70% of these databases were not downloaded or were only available upon request from the authors. This makes it difficult for users to assess data quality and integrity, in addition to causing limited reuse. Current studies also have a bias (>50%) toward bitter and sweet molecules compared to other sensations. As a result, other taste sensations have received less attention. This highlights the need to further annotate molecules with tastes such as sour, salty, and spicy in publications and experimental records. Furthermore, the content of most flavor molecules from natural resources is unavailable in any databases, which may limit their application in the industry.

Based on these data, molecular simulation and machine learning have been widely used to identify novel flavor molecules. Multiple types of data (e.g., molecule structures of flavor molecules and features extracted from mass spectra) and algorithms (e.g., RF, SVM, and CNN) have been used for ML modeling. These models help prioritize a large number of compounds in terms of their desired flavor properties as an *in silico* methodology, in turn significantly reducing the number of candidate chemicals for detailed sensory analyses. The feasibility and efficiency of ML modeling are widely accepted; however, issues with untimely updates, data inaccessibility, and code nondisclosure still remain. Therefore, we strongly encourage authors to make all data and code openly accessible during the publication process in future studies. Finally, we discussed the limitations and lack of current knowledge associated with poor coverage of known molecules and highlighted the future computational strategies for identifying novel flavor molecules. By harnessing the power of artificial intelligence and utilizing the wealth of multi-omics data, we will be able to uncover novel flavor compounds and gain a deeper understanding of the intricate interplay between molecules that shape our perceptions of taste and aroma. This could pave the way for the creation of innovative food products with rich flavor profiles and enhanced nutritional value. In future work, we will propose an impartial evaluation system for flavor molecule databases according to their data quality, availability, and transparency to advance findable, accessible, interoperable, and reusable research.

## Data Availability

To facilitate further usage, we provide flavor molecule data collected from publicly available databases in a GitHub repository: https://github.com/DachuanZhang-FutureFood/flavor-science.

## Abbreviations Used

MS: molecular simulation
ML: machine learning
CAS: Chemical Abstract Service
URL: uniform resource locator
QSAR: quantitative conformational relationship
VCF: volatile compounds in food
RF: random forest
SVM: support vector machine
CNN: convolutional neural network
T1R1/T1R3: taste receptor type 1 members 1/3
PKD1L3: polycystin 1 like 3
cAMP: cyclic AMP
OBP1: odorant binding protein 1
BGCs: biosynthetic gene clusters
pHMMs: profile hidden Markov models

## References

[ref1] Wilk K.; Korytek W.; Pelczyńska M.; Moszak M.; Bogdański P. The Effect of Artificial Sweeteners Use on Sweet Taste Perception and Weight Loss Efficacy: A Review. Nutrients 2022, 14 (6), 1261 10.3390/nu14061261. 35334918 PMC8954878

[ref2] Eelager M. P.; Masti S. P.; Chougale R. B.; Hiremani V. D.; Narasgoudar S. S.; Dalbanjan N. P.; S.K. P. K. Evaluation of mechanical, antimicrobial, and antioxidant properties of vanillic acid induced chitosan/poly (vinyl alcohol) active films to prolong the shelf life of green chilli. Int. J. Biol. Macromol. 2023, 232, 123499 10.1016/j.ijbiomac.2023.123499. 36736522

[ref3] Smutzer G.; Cherian S.; Patel D.; Lee B. S.; Lee K.; Sotelo A. R.; Mitchell K.-D. W. A formulation for suppressing bitter taste in the human oral cavity. Physiology & Behavior 2020, 226, 113129 10.1016/j.physbeh.2020.113129. 32791180

[ref4] Zhang D.; Ouyang S.; Cai M.; Zhang H.; Ding S.; Liu D.; Cai P.; Le Y.; Hu Q. N. FADB-China: A molecular-level food adulteration database in China based on molecular fingerprints and similarity algorithms prediction expansion. Food Chem. 2020, 327, 127010 10.1016/j.foodchem.2020.127010. 32442849

[ref5] Zhang D.; Cheng X.; Sun D.; Ding S.; Cai P.; Yuan L.; Tian Y.; Tu W.; Hu Q.-N. AdditiveChem: A comprehensive bioinformatics knowledge-base for food additive chemicals. Food Chem. 2020, 308, 125519 10.1016/j.foodchem.2019.125519. 31648087

[ref6] Chassaing B.; Koren O.; Goodrich J. K.; Poole A. C.; Srinivasan S.; Ley R. E.; Gewirtz A. T. Dietary emulsifiers impact the mouse gut microbiota promoting colitis and metabolic syndrome. Nature 2015, 519 (7541), 92–96. 10.1038/nature14232. 25731162 PMC4910713

[ref7] Curwin B. D.; Deddens J. A.; McKernan L. T. Flavoring exposure in food manufacturing. J. Expo Sci. Environ. Epidemiol 2015, 25 (3), 324–333. 10.1038/jes.2014.52. 25052692 PMC4520397

[ref8] Chandra S.; Qureshi S.; Chopra D.; Shukla S.; Patel S. K.; Singh J.; Ray R. S. UVR-induced phototoxicity mechanism of methyl N-methylanthranilate in human keratinocyte cell line. Toxicology in Vitro 2022, 80, 105322 10.1016/j.tiv.2022.105322. 35085765

[ref9] Cohen S. M.; Eisenbrand G.; Fukushima S.; Gooderham N. J.; Guengerich F. P.; Hecht S. S.; Rietjens I. M. C. M.; Bastaki M.; Davidsen J. M.; Harman C. L.; et al. FEMA GRAS assessment of natural flavor complexes: Citrus-derived flavoring ingredients. Food Chem. Toxicol. 2019, 124, 192–218. 10.1016/j.fct.2018.11.052. 30481573

[ref10] Garg N.; Sethupathy A.; Tuwani R.; Rakhi N. K.; Dokania S.; Iyer A.; Gupta A.; Agrawal S.; Singh N.; Shukla S.; et al. FlavorDB: a database of flavor molecules. Nucleic Acids Res. 2018, 46 (D1), D1210–D1216. 10.1093/nar/gkx957. 29059383 PMC5753196

[ref11] Marvin H. J.; Janssen E. M.; Bouzembrak Y.; Hendriksen P. J.; Staats M. Big data in food safety: An overview. Crit Rev. Food Sci. Nutr 2017, 57 (11), 2286–2295. 10.1080/10408398.2016.1257481. 27819478

[ref12] Tuwani R.; Wadhwa S.; Bagler G. BitterSweet: Building machine learning models for predicting the bitter and sweet taste of small molecules. Sci. Rep 2019, 9, 13 10.1038/s41598-019-43664-y. 31073241 PMC6509165

[ref13] Loss A.; Bunsmann P.; Bohne A.; Loss A.; Schwarzer E.; Lang E.; von der Lieth C. W. SWEET-DB: an attempt to create annotated data collections for carbohydrates. Nucleic Acids Res. 2002, 30 (1), 405–408. 10.1093/nar/30.1.405. 11752350 PMC99123

[ref14] Ahmed J.; Preissner S.; Dunkel M.; Worth C. L.; Eckert A.; Preissner R. SuperSweet—a resource on natural and artificial sweetening agents. Nucleic Acids Res. 2011, 39, D377–D382. 10.1093/nar/gkq917. 20952410 PMC3013782

[ref15] Chéron J. B.; Casciuc I.; Golebiowski J.; Antonczak S.; Fiorucci S. Sweetness prediction of natural compounds. Food Chem. 2017, 221, 1421–1425. 10.1016/j.foodchem.2016.10.145. 27979110

[ref16] Zheng S. Q.; Chang W. P.; Xu W. X.; Xu Y.; Lin F. e-Sweet: A Machine-Learning Based Platform for the Prediction of Sweetener and Its Relative Sweetness. Front. Chem. 2019, 7, 14 10.3389/fchem.2019.00035. 30761295 PMC6363693

[ref17] Rodgers S.; Busch J.; Peters H.; Christ-Hazelhof E. Building a tree of knowledge: analysis of bitter molecules. Chem. Senses 2005, 30 (7), 547–557. 10.1093/chemse/bji048. 16079246

[ref18] Wiener A.; Shudler M.; Levit A.; Niv M. Y. BitterDB: a database of bitter compounds. Nucleic Acids Res. 2012, 40 (D1), D413–D419. 10.1093/nar/gkr755. 21940398 PMC3245057

[ref19] Dagan-Wiener A.; Di Pizio A.; Nissim I.; Bahia M. S.; Dubovski N.; Margulis E.; Niv M. Y. BitterDB: taste ligands and receptors database in 2019. Nucleic Acids Res. 2019, 47 (D1), D1179–D1185. 10.1093/nar/gky974. 30357384 PMC6323989

[ref20] Bayer S.; Mayer A. I.; Borgonovo G.; Morini G.; Di Pizio A.; Bassoli A. Chemoinformatics View on Bitter Taste Receptor Agonists in Food. J. Agric. Food Chem. 2021, 69 (46), 13916–13924. 10.1021/acs.jafc.1c05057. 34762411 PMC8630789

[ref21] Zheng S. Q.; Jiang M. Y.; Zhao C. W.; Zhu R.; Hu Z. C.; Xu Y.; Lin F. e-Bitter: Bitterant Prediction by the Consensus Voting From the Machine-Learning Methods. Front. Chem. 2018, 6, 18 10.3389/fchem.2018.00082. 29651416 PMC5885771

[ref22] Bai G. L.; Wu T. T.; Zhao L. B.; Wang X. L.; Li S.; Ni X. CBDPS 1.0: A Python GUI Application for Machine Learning Models to Predict Bitter-Tasting Children’s Oral Medicines. Chem. Pharm. Bull. 2021, 69 (10), 989–994. 10.1248/cpb.c20-00866. 34421065

[ref23] Dragos D.; Gilca M. PhytoMolecularTasteDB: An integrative database on the “molecular taste” of Indian medicinal plants. Data in Brief 2018, 19, 1237–1241. 10.1016/j.dib.2018.04.048. 30246068 PMC6141601

[ref24] Rojas C.; Ballabio D.; Pacheco Sarmiento K.; Pacheco Jaramillo E.; Mendoza M.; García F. ChemTastesDB: A curated database of molecular tastants. Food Chemistry: Molecular Sciences 2022, 4, 100090 10.1016/j.fochms.2022.100090. 35415670 PMC8991844

[ref25] Gradinaru T. C.; Petran M.; Dragos D.; Gilca M. PlantMolecularTasteDB: A Database of Taste Active Phytochemicals. Front. Pharmacol. 2022, 12, 6 10.3389/fphar.2021.751712. PMC8789873 35095484

[ref26] Arn H.; Acree T. Flavornet: A database of aroma compounds based on odor potency in natural products. Developments in food science 1998, 40, 27–28. 10.1016/S0167-4501(98)80029-0.

[ref27] Dunkel M.; Schmidt U.; Struck S.; Berger L.; Gruening B.; Hossbach J.; Jaeger I. S.; Effmert U.; Piechulla B.; Eriksson R.; et al. SuperScent - A database of flavors and scents. Nucleic Acids Res. 2009, 37, D291–D294. 10.1093/nar/gkn695. 18931377 PMC2686498

[ref28] Kumar R.; Kaur R.; Auffarth B.; Bhondekar A. P. Understanding the Odour Spaces: A Step towards Solving Olfactory Stimulus-Percept Problem. PLoS One 2015, 10 (10), e0141263 10.1371/journal.pone.0141263. 26484763 PMC4615634

[ref29] Ueda Y.; Itoh M. Database of Pesticides and Off-flavors for Health Crisis Management. Food Hyg. Saf. Sci. 2016, 57 (2), 46–50. 10.3358/shokueishi.57.46. 27211918

[ref30] Kumar Y.; Prakash O.; Tripathi H.; Tandon S.; Gupta M. M.; Rahman L. U.; Lal R. K.; Semwal M.; Darokar M. P.; Khan F. AromaDb: A Database of Medicinal and Aromatic Plant’s Aroma Molecules With Phytochemistry and Therapeutic Potentials. Frontiers in Plant Science 2018, 9, 11 10.3389/fpls.2018.01081. 30150996 PMC6099104

[ref31] Sharma A.; Saha B. K.; Kumar R.; Varadwaj P. K. OlfactionBase: a repository to explore odors, odorants, olfactory receptors and odorant-receptor interactions. Nucleic Acids Res. 2022, 50 (D1), D678–D686. 10.1093/nar/gkab763. 34469532 PMC8728123

[ref32] Vidal-Limon A.; Aguilar-Toalá J. E.; Liceaga A. M. Integration of Molecular Docking Analysis and Molecular Dynamics Simulations for Studying Food Proteins and Bioactive Peptides. J. Agric. Food Chem. 2022, 70 (4), 934–943. 10.1021/acs.jafc.1c06110. 34990125

[ref33] Acevedo W.; González-Nilo F.; Agosin E. Docking and Molecular Dynamics of Steviol Glycoside–Human Bitter Receptor Interactions. J. Agric. Food Chem. 2016, 64 (40), 7585–7596. 10.1021/acs.jafc.6b02840. 27640213

[ref34] Ryckaert J.-P.; Ciccotti G.; Berendsen H. J. C. Numerical integration of the cartesian equations of motion of a system with constraints: molecular dynamics of n-alkanes. J. Comput. Phys. 1977, 23 (3), 327–341. 10.1016/0021-9991(77)90098-5.

[ref35] Yu Y.; Xu S.; He R.; Liang G. Application of Molecular Simulation Methods in Food Science: Status and Prospects. J. Agric. Food Chem. 2023, 71 (6), 2684–2703. 10.1021/acs.jafc.2c06789. 36719790

[ref36] Pinzi L.; Rastelli G. Molecular Docking: Shifting Paradigms in Drug Discovery. Int. J. Mol. Sci. 2019, 20 (18), 4331 10.3390/ijms20184331. 31487867 PMC6769923

[ref37] van Gunsteren W. F.; Dolenc J.; Mark A. E. Molecular simulation as an aid to experimentalists. Curr. Opin. Struct. Biol. 2008, 18 (2), 149–153. 10.1016/j.sbi.2007.12.007. 18280138

[ref38] Zehentner S.; Reiner A. T.; Grimm C.; Somoza V. The Role of Bitter Taste Receptors in Cancer: A Systematic Review. Cancers 2021, 13 (23), 5891 10.3390/cancers13235891. 34885005 PMC8656863

[ref39] Ishimaru Y.; Inada H.; Kubota M.; Zhuang H.; Tominaga M.; Matsunami H. Transient receptor potential family members PKD1L3 and PKD2L1 form a candidate sour taste receptor. Proc. Natl. Acad. Sci. U. S. A. 2006, 103 (33), 12569–12574. 10.1073/pnas.0602702103. 16891422 PMC1531643

[ref40] Nomura K.; Nakanishi M.; Ishidate F.; Iwata K.; Taruno A. All-Electrical Ca^2+^-Independent Signal Transduction Mediates Attractive Sodium Taste in Taste Buds. Neuron 2020, 106 (5), 816–829. 10.1016/j.neuron.2020.03.006. 32229307

[ref41] Keller A.; Gerkin R. C.; Guan Y. F.; Dhurandhar A.; Turu G.; Szalai B.; Mainland J. D.; Ihara Y.; Yu C. W.; Wolfinger R.; et al. Predicting human olfactory perception from chemical features of odor molecules. Science 2017, 355 (6327), 820 10.1126/science.aal2014. 28219971 PMC5455768

[ref42] Villar P. S.; Delgado R.; Vergara C.; Reyes J. G.; Bacigalupo J. Energy Requirements of Odor Transduction in the Chemosensory Cilia of Olfactory Sensory Neurons Rely on Oxidative Phosphorylation and Glycolytic Processing of Extracellular Glucose. J. Neurosci. 2017, 37 (23), 5736–5743. 10.1523/JNEUROSCI.2640-16.2017. 28500222 PMC6596473

[ref43] Miao Y. L.; Ni H.; Zhang X. Y.; Zhi F. D.; Long X.; Yang X. P.; He X.; Zhang L. J. Investigating mechanism of sweetness intensity differences through dynamic analysis of sweetener-T1R2-membrane systems. Food Chem. 2022, 374, 131807 10.1016/j.foodchem.2021.131807. 34915374

[ref44] Jang J.; Kim S. K.; Guthrie B.; Goddard W. A. Synergic Effects in the Activation of the Sweet Receptor GPCR Heterodimer for Various Sweeteners Predicted Using Molecular Metadynamics Simulations. J. Agric. Food Chem. 2021, 69 (41), 12250–12261. 10.1021/acs.jafc.1c03779. 34613740

[ref45] Acevedo W.; Ramirez-Sarmiento C. A.; Agosin E. Identifying the interactions between natural, non-caloric sweeteners and the human sweet receptor by molecular docking. Food Chem. 2018, 264, 164–171. 10.1016/j.foodchem.2018.04.113. 29853362

[ref46] Acevedo W.; Gonzalez-Nilo F.; Agosin E. Docking and Molecular Dynamics of Steviol Glycoside-Human Bitter Receptor Interactions. J. Agric. Food Chem. 2016, 64 (40), 7585–7596. 10.1021/acs.jafc.6b02840. 27640213

[ref47] Zhang Y.; Gao X. C.; Pan D. D.; Zhang Z. G.; Zhou T. Q.; Dang Y. L. Isolation, characterization and molecular docking of novel umami and umami-enhancing peptides from Ruditapes philippinarum. Food Chem. 2021, 343, 128522 10.1016/j.foodchem.2020.128522. 33208237

[ref48] Gao B.; Hu X.; Xue H.; Li R.; Liu H.; Han T.; Ruan D.; Tu Y.; Zhao Y. Isolation and screening of umami peptides from preserved egg yolk by nano-HPLC-MS/MS and molecular docking. Food Chem. 2022, 377, 131996 10.1016/j.foodchem.2021.131996. 34998156

[ref49] do Bomfim M. R.; Araújo J. S. C.; Macêdo W. J. d. C.; Santos C. B. R. d.; Leite F. H. A. Identification of potential modulator of Anopheles gambiae odorant binding protein 1 by hierarchical virtual screening and molecular dynamics. J. Biomol. Struct. Dyn. 2021, 39 (16), 6031–6043. 10.1080/07391102.2020.1796807. 32696721

[ref50] Han M.; Liu S.; Zhang D.; Zhang R.; Liu D.; Xing H.; Sun D.; Gong L.; Cai P.; Tu W. AddictedChem: A Data-Driven Integrated Platform for New Psychoactive Substance Identification. Molecules 2022, 27 (12), 3931 10.3390/molecules27123931. 35745053 PMC9227411

[ref51] Achary P. G. R.; Toropova A. P.; Toropov A. A. Combinations of graph invariants and attributes of simplified molecular input-line entry system (SMILES) to build up models for sweetness. Food Research International 2019, 122, 40–46. 10.1016/j.foodres.2019.03.067. 31229093

[ref52] Barker J. S.; Hattotuwagama C. K.; Drew M. G. B. Computational studies of sweet-tasting molecules. Pure Appl. Chem. 2002, 74 (7), 1207–1217. 10.1351/pac200274071207.

[ref53] Zhong M.; Chong Y.; Nie X.; Yan A.; Yuan Q. Prediction of Sweetness by Multilinear Regression Analysis and Support Vector Machine. J. Food Sci. 2013, 78 (9), S1445–S1450. 10.1111/1750-3841.12199. 23915005

[ref54] Goel A.; Gajula K.; Gupta R.; Rai B. In-silico prediction of sweetness using structure-activity relationship models. Food Chem. 2018, 253, 127–131. 10.1016/j.foodchem.2018.01.111. 29502811

[ref55] Bouysset C.; Belloir C.; Antonczak S.; Briand L.; Fiorucci S. Novel scaffold of natural compound eliciting sweet taste revealed by machine learning. Food Chem. 2020, 324, 126864 10.1016/j.foodchem.2020.126864. 32344344

[ref56] Margulis E.; Dagan-Wiener A.; Ives R. S.; Jaffari S.; Siems K.; Niv M. Y. Intense bitterness of molecules: Machine learning for expediting drug discovery. Comp. Struct. Biotechnol. J.. 2021, 19, 568–576. 10.1016/j.csbj.2020.12.030. PMC7807207 33510862

[ref57] Zhang L.; Mao H.; Liu L.; Du J.; Gani R. A machine learning based computer-aided molecular design/screening methodology for fragrance molecules. Comput. Chem. Eng. 2018, 115, 295–308. 10.1016/j.compchemeng.2018.04.018.

[ref58] Dagan-Wiener A.; Nissim I.; Ben Abu N.; Borgonovo G.; Bassoli A.; Niv M. Y. Bitter or not? BitterPredict, a tool for predicting taste from chemical structure. Sci. Rep 2017, 7 (1), 12074 10.1038/s41598-017-12359-7. 28935887 PMC5608695

[ref59] Licon C. C.; Bosc G.; Sabri M.; Mantel M.; Fournel A.; Bushdid C.; Golebiowski J.; Robardet C.; Plantevit M.; Kaytoue M.; et al. Chemical features mining provides new descriptive structure-odor relationships. PLoS Comput. Biol. 2019, 15 (4), e1006945 10.1371/journal.pcbi.1006945. 31022180 PMC6504111

[ref60] Rojas C.; Ballabio D.; Consonni V.; Tripaldi P.; Mauri A.; Todeschini R. Quantitative structure-activity relationships to predict sweet and non-sweet tastes. Theor. Chem. Acc. 2016, 135 (3), 13 10.1007/s00214-016-1812-1.

[ref61] Banerjee P.; Preissner R. BitterSweet Forest: A Random Forest Based Binary Classifier to Predict Bitterness and Sweetness of Chemical Compounds. Front. Chem. 2018, 6, 10 10.3389/fchem.2018.00093. 29696137 PMC5905275

[ref62] Fritz F.; Preissner R.; Banerjee P. VirtualTaste: a web server for the prediction of organoleptic properties of chemical compounds. Nucleic Acids Res. 2021, 49 (W1), W679–W684. 10.1093/nar/gkab292. 33905509 PMC8262722

[ref63] Chacko R.; Jain D.; Patwardhan M.; Puri A.; Karande S.; Rai B. Data based predictive models for odor perception. Sci. Rep 2020, 10 (1), 13 10.1038/s41598-020-73978-1. 33051564 PMC7553929

[ref64] Nozaki Y.; Nakamoto T. Odor Impression Prediction from Mass Spectra. PLoS One 2016, 11 (6), e0157030 10.1371/journal.pone.0157030. 27326765 PMC4915715

[ref65] Nozaki Y.; Nakamoto T. Predictive modeling for odor character of a chemical using machine learning combined with natural language processing. PLoS One 2018, 13 (6), e0198475 10.1371/journal.pone.0198475. 29902194 PMC6002022

[ref66] Jiang J. C.; Lin X. X.; Jiang Y. Q.; Jiang L. Z.; Lv Z. B. Identify Bitter Peptides by Using Deep Representation Learning Features. Int. J. Mol. Sci. 2022, 23 (14), 7877 10.3390/ijms23147877. 35887225 PMC9315524

[ref67] Temussi P. A. New Insights into the Characteristics of Sweet and Bitter Taste Receptors. In International Review of Cell and Molecular Biology; Jeon K. W., Ed.; Academic Press, 2011; Vol. 291, pp 191–226. 10.1016/B978-0-12-386035-4.00006-9 22017977

[ref68] Li X.; Luo D. H.; Cheng Y.; Wong K. Y.; Hung K. Identifying the Primary Odor Perception Descriptors by Multi-Output Linear Regression Models. Appl. Sci.-Basel 2021, 11 (8), 3320 10.3390/app11083320.

[ref69] Goel A.; Gajula K.; Gupta R.; Rai B. In-silico screening of database for finding potential sweet molecules: A combined data and structure based modeling approach. Food Chem. 2021, 343, 128538 10.1016/j.foodchem.2020.128538. 33183872

[ref70] Xiu H. X.; Liu Y. J.; Yang H. H.; Ren H. B.; Luo B. W.; Wang Z. P.; Shao H.; Wang F. Z.; Zhang J. J.; Wang Y. T. Identification of novel umami molecules via QSAR models and molecular docking. Food Funct. 2022, 13 (14), 7529–7539. 10.1039/D2FO00544A. 35765918

[ref71] Gentile F.; Agrawal V.; Hsing M.; Ton A.-T.; Ban F.; Norinder U.; Gleave M. E.; Cherkasov A. Deep Docking: A Deep Learning Platform for Augmentation of Structure Based Drug Discovery. ACS Central Science 2020, 6 (6), 939–949. 10.1021/acscentsci.0c00229. 32607441 PMC7318080

[ref72] Armstrong D. R.; Berrisford J. M.; Conroy M. J.; Gutmanas A.; Anyango S.; Choudhary P.; Clark A. R.; Dana J. M.; Deshpande M.; Dunlop R.; et al. PDBe: improved findability of macromolecular structure data in the PDB. Nucleic Acids Res. 2019, 48 (D1), D335–D343. 10.1093/nar/gkz990. PMC7145656 31691821

[ref73] Baek M.; DiMaio F.; Anishchenko I.; Dauparas J.; Ovchinnikov S.; Lee G. R.; Wang J.; Cong Q.; Kinch L. N.; Schaeffer R. D.; et al. Accurate prediction of protein structures and interactions using a three-track neural network. Science 2021, 373 (6557), 871–876. 10.1126/science.abj8754. 34282049 PMC7612213

[ref74] Jumper J.; Evans R.; Pritzel A.; Green T.; Figurnov M.; Ronneberger O.; Tunyasuvunakool K.; Bates R.; Žídek A.; Potapenko A.; et al. Highly accurate protein structure prediction with AlphaFold. Nature 2021, 596 (7873), 583–589. 10.1038/s41586-021-03819-2. 34265844 PMC8371605

[ref75] Lundberg S.; Lee S.-I. A Unified Approach to Interpreting Model Predictions. Advances in Neural Information Processing Systems 30 (NIPS 2017), Long Beach, CA, December 4–9, 2017.

[ref76] Ouyang R.; Curtarolo S.; Ahmetcik E.; Scheffler M.; Ghiringhelli L. M. SISSO: A compressed-sensing method for identifying the best low-dimensional descriptor in an immensity of offered candidates. Phys. Rev. Mater. 2018, 2 (8), 083802 10.1103/PhysRevMaterials.2.083802.

[ref77] Guo T.; Pan F.; Cui Z.; Yang Z.; Chen Q.; Zhao L.; Song H. FAPD: An Astringency Threshold and Astringency Type Prediction Database for Flavonoid Compounds Based on Machine Learning. J. Agric. Food Chem. 2023, 71 (9), 4172–4183. 10.1021/acs.jafc.2c08822. 36825752

[ref78] Tian Y.; Wang X.; Yao X.; Liu H.; Yang Y. Predicting molecular properties based on the interpretable graph neural network with multistep focus mechanism. Briefings in Bioinformatics 2023, 10.1093/bib/bbac534. 36526280

[ref79] Zhang D.; Gong L.; Ding S.; Tian Y.; Jia C.; Liu D.; Han M.; Cheng X.; Sun D.; Cai P.; et al. FRCD: A comprehensive food risk component database with molecular scaffold, chemical diversity, toxicity, and biodegradability analysis. Food Chem. 2020, 318, 126470 10.1016/j.foodchem.2020.126470. 32120139

[ref80] Sorokina M.; Merseburger P.; Rajan K.; Yirik M. A.; Steinbeck C. COCONUT online: Collection of Open Natural Products database. J. Cheminformatics 2021, 13 (1), 13 10.1186/s13321-020-00478-9. PMC7798278 33423696

[ref81] Banerjee P.; Erehman J.; Gohlke B. O.; Wilhelm T.; Preissner R.; Dunkel M. Super Natural II-a database of natural products. Nucleic Acids Res. 2015, 43 (D1), D935–D939. 10.1093/nar/gku886. 25300487 PMC4384003

[ref82] Tian Y.; Wu L.; Yuan L.; Ding S.; Chen F.; Zhang T.; Ren A.; Zhang D.; Tu W.; Chen J.; et al. BCSExplorer: a customized biosynthetic chemical space explorer with multifunctional objective function analysis. Bioinformatics 2019, 36 (5), 1642–1643. 10.1093/bioinformatics/btz755. 31593245

[ref83] Medema M. H.; de Rond T.; Moore B. S. Mining genomes to illuminate the specialized chemistry of life. Nat. Rev. Genet. 2021, 22 (9), 553–571. 10.1038/s41576-021-00363-7. 34083778 PMC8364890

[ref84] Blin K.; Shaw S.; Kloosterman A. M; Charlop-Powers Z.; van Wezel G. P; Medema M. H; Weber T. antiSMASH 6.0: improving cluster detection and comparison capabilities. Nucleic Acids Res. 2021, 49 (W1), W29–W35. 10.1093/nar/gkab335. 33978755 PMC8262755

[ref85] Skinnider M. A.; Johnston C. W.; Gunabalasingam M.; Merwin N. J.; Kieliszek A. M.; MacLellan R. J.; Li H.; Ranieri M. R. M.; Webster A. L. H.; Cao M. P. T.; et al. Comprehensive prediction of secondary metabolite structure and biological activity from microbial genome sequences. Nat. Commun. 2020, 11 (1), 6058 10.1038/s41467-020-19986-1. 33247171 PMC7699628

[ref86] Hannigan G. D.; Prihoda D.; Palicka A.; Soukup J.; Klempir O.; Rampula L.; Durcak J.; Wurst M.; Kotowski J.; Chang D.; et al. A deep learning genome-mining strategy for biosynthetic gene cluster prediction. Nucleic Acids Res. 2019, 47 (18), 13 10.1093/nar/gkz654. PMC6765103 31400112

[ref87] Schwab W.; Davidovich-Rikanati R.; Lewinsohn E. Biosynthesis of plant-derived flavor compounds. Plant J. 2008, 54 (4), 712–732. 10.1111/j.1365-313X.2008.03446.x. 18476874

[ref88] Kautsar S. A.; Suarez Duran H. G.; Blin K.; Osbourn A.; Medema M. H. plantiSMASH: automated identification, annotation and expression analysis of plant biosynthetic gene clusters. Nucleic Acids Res. 2017, 45 (W1), W55–W63. 10.1093/nar/gkx305. 28453650 PMC5570173

[ref89] Hjorleifsson Eldjarn G.; Ramsay A.; van der Hooft J. J. J.; Duncan K. R.; Soldatou S.; Rousu J.; Daly R.; Wandy J.; Rogers S. Ranking microbial metabolomic and genomic links in the NPLinker framework using complementary scoring functions. PLOS Computational Biology 2021, 17 (5), e1008920 10.1371/journal.pcbi.1008920. 33945539 PMC8130963

[ref90] Walters W. P.; Barzilay R. Applications of Deep Learning in Molecule Generation and Molecular Property Prediction. Acc. Chem. Res. 2021, 54 (2), 263–270. 10.1021/acs.accounts.0c00699. 33370107

[ref91] Bilodeau C.; Jin W.; Jaakkola T.; Barzilay R.; Jensen K. F. Generative models for molecular discovery: Recent advances and challenges. WIREs Computational Molecular Science 2022, 12 (5), e1608 10.1002/wcms.1608.

[ref92] Zhavoronkov A.; Ivanenkov Y. A.; Aliper A.; Veselov M. S.; Aladinskiy V. A.; Aladinskaya A. V.; Terentiev V. A.; Polykovskiy D. A.; Kuznetsov M. D.; Asadulaev A.; et al. Deep learning enables rapid identification of potent DDR1 kinase inhibitors. Nat. Biotechnol. 2019, 37 (9), 1038–1040. 10.1038/s41587-019-0224-x. 31477924

[ref93] Skinnider M. A.; Wang F.; Pasin D.; Greiner R.; Foster L. J.; Dalsgaard P. W.; Wishart D. S. A deep generative model enables automated structure elucidation of novel psychoactive substances. Nature Machine Intelligence 2021, 3 (11), 973–984. 10.1038/s42256-021-00407-x.

